# Implementation of a method for sperm cryopreservation in sceloporine lizards

**DOI:** 10.1093/conphys/coac068

**Published:** 2022-11-09

**Authors:** Uriel Á Sánchez-Rivera, Alfredo Medrano, Norma B Cruz-Cano, Alicia Alcántar-Rodríguez, Rodrigo Dávila-Govantes, Yabín J Castro-Camacho, Martín Martínez-Torres

**Affiliations:** Laboratorio de Biología de la Reproducción. Facultad de Estudios Superiores Iztacala, Universidad Nacional Autónoma de México, 54090, México; Laboratorio de Reproducción Animal. Facultad de Estudios Superiores Cuautitlán, Universidad Nacional Autónoma de México, 54714, México; Posgrado en Ciencias de la Producción y de la Salud Animal, Universidad Nacional Autónoma de México, 04510, México; Laboratorio de Reproducción Animal. Facultad de Estudios Superiores Cuautitlán, Universidad Nacional Autónoma de México, 54714, México; Laboratorio de Biología de la Reproducción. Facultad de Estudios Superiores Iztacala, Universidad Nacional Autónoma de México, 54090, México; Laboratorio de Reproducción Animal. Facultad de Estudios Superiores Cuautitlán, Universidad Nacional Autónoma de México, 54714, México; Laboratorio de Biología de la Reproducción. Facultad de Estudios Superiores Iztacala, Universidad Nacional Autónoma de México, 54090, México; Laboratorio de Biología de la Reproducción. Facultad de Estudios Superiores Iztacala, Universidad Nacional Autónoma de México, 54090, México; Laboratorio de Biología de la Reproducción. Facultad de Estudios Superiores Iztacala, Universidad Nacional Autónoma de México, 54090, México

**Keywords:** assisted reproductive techniques, cryopreservation, cryoprotectant, glycerol, reptile, semen

## Abstract

Actual loss of lizard biodiversity continues, even with the implementation of conventional conservation programs. An approach including assisted reproductive techniques such as sperm cryopreservation may contribute to the management of endangered species. We developed a method for sperm cryopreservation in sceloporine lizards and compared the response among the studied species. Prior to the mating season, we obtained semen from adult males of *Sceloporus aeneus* (*n* = 21), *Sceloporus grammicus* (*n* = 20) and *Sceloporus torquatus* (*n* = 21) via pressure of the genital papilla. Volume and sperm concentration were measured before semen dilution in a Tris–egg yolk (TEY) medium to evaluate progressive motility, sperm viability, morphology, plasma membrane and acrosome integrity. Then, we cooled the remaining volumes to 5°C at a rate of 0.1°C per minute to incorporate glycerol (8% v/v) in two fractions. Immediately afterwards, we placed 40 μl of the mix on solid CO_2_ to form pellets and immersed them in liquid nitrogen for storage. We thawed the pellets at 29°C for 3 minutes and diluted them 1:1 (v/v) in TEY medium to assess sperm quality. We found a positive relationship between body weight and seminal volume in *S. grammicus* and *S. torquatus* and a negative correlation with sperm concentration in *S. grammicus* (*P* < 0.05). Moreover, we observed that the freezing–thawing process decreased sperm quality in the three species, mostly affecting motility and viability. However, *S. torquatus* and *S. aeneus* showed a higher sperm tolerance than *S. grammicus*.

## Teaser

We developed a protocol for sperm cryopreservation in sceloporine lizards using Tris-egg yolk medium supplemented with 8% glycerol. We found that the freeze‒thaw process diminished sperm quality in the three species investigated (Sceloporus aeneus, S. grammicus, and S. torquatus). However, S. grammicus showed the lowest sperm cryoresistance of the species studied.

## Introduction

The diversity of herpetofauna has declined in recent decades due to anthropogenic effects (Sinervo *et al.*, 2010; Stapley *et al.*, 2015; Oliver *et al.*, 2018; Diele-Viegas *et al.*, 2019; Wiens *et al.*, 2019). Although it is accepted that one-third of lizard species are in any threat category (Young *et al.*, 2017), this situation may be underestimated (Fitzgerald *et al.*, 2018). Some efforts try to counteract the loss of reptile biodiversity, but they remain insufficient (Vitt and Caldwell, 2014; Roll *et al.*, 2017). The implementation of assisted reproduction technologies (ARTs) may be an excellent opportunity to improve conservation programs. ARTs are widely used in domestic (Cheng *et al.*, 1986; Fukuda *et al.*, 1990; Nagai, 1996; Pope *et al.*, 1997; Sánchez and Rubilar, 2001) and wild species (Shivaji *et al.*, 2003; Hermes *et al.*, 2009), but their development in lizards is still incipient (Clulow and Clulow, 2016; Martínez-Torres *et al.*, 2019b; Perry *et al.*, 2021).

Several methods have been developed for semen collection, such as electroejaculation (Zimmerman *et al.*, 2013; López-Juri *et al.*, 2018; Perry *et al.*, 2019; Martínez-Torres *et al.*, 2019a; Mason *et al.*, 2021), ventral massages (Molinia *et al.*, 2010; Kahrl and Cox, 2017) and pressure on the genital papilla (Martínez-Torres *et al.*, 2019b). Some studies have included analysis of sperm quality (López-Juri *et al.*, 2018; Martínez-Torres *et al.*, 2019b; Mason *et al.*, 2021), but sperm cryopreservation has made little progress in this group (Young *et al.*, 2017; Campbell *et al.*, 2020, 2021; Hobbs *et al.*, 2021).

We think that the importance of sperm cryopreservation lies in the possibility of extending the reproductive potential of individuals and preserving male fertility in germplasm banks. However, before its application in threatened species, the implementation of this methodology by applying non-destructive methods to obtain semen (Hobbs *et al.*, 2021) in non-endangered species is needed.

Sceloporine lizards are frequently used in different research topics, such as reproductive biology, ecology, genetics and systematics. Despite the decline or loss of some of their populations (Sinervo *et al.*, 2010), there are several species that may be adequate models to develop ARTs since they are abundant, widely distributed and considered to be of the least concern (IUCN, 2021). However, the low seminal volumes obtained in small- and medium-sized species represent a challenge in designing a method with great sperm recovery after freeze–thaw processes.

For this study, we tried Tris–egg yolk (TEY), since it is a widely used extender for sperm cryopreservation (Cabrera *et al.*, 2005; Kulaksız *et al.*, 2010) and secondly for its protective effect on sperm membranes (mainly against cold shock) due to the contribution to low-density lipoproteins (Benson *et al.*, 2012; Swelum *et al.*, 2018). On the other hand, glycerol is one of the least toxic and most effective cryoprotectants (Hammerstedt and Graham, 1992; Prieto *et al.*, 2014) since it allows preservation of sperm motility and membrane integrity. Although it has been tested in some lizards (Young *et al.*, 2017; Campbell *et al.*, 2021; Hobbs *et al.*, 2021), their protective response must be evaluated before being discarded. This study aimed to implement a method for sperm cryopreservation in small- (*S. aeneus* and *S. grammicus*) and medium-sized (*S. torquatus*) lizards and compare their tolerance to the freezing protocol.

## Materials and Methods

### Animals

We captured 62 adult males of sceloporine lizards at different locations in the State of Mexico: *S. aeneus* (*n* = 21) in Tlazala de Isidro Fabela (19° 55’ N, 99° 41’ W, 2793 m altitude), *S. grammicus* (*n* = 20) in the School of Higher Studies Iztacala (FESI) of the National Autonomous University of Mexico (UNAM), Tlalnepantla (19° 52’ N, 99°18’ W, 2246 m altitude) and *S. torquatus* (*n* = 21) in the Sierra de Guadalupe State Park, Coacalco (19° 61’ N, 99° 11’ W, 2480 m altitude). The collections were made from 2018 to 2019 before the mating season for each species (March–April, July–August and October–November, respectively) under the scientific collecting license SGPA/DGVS/02921/19 granted by the Secretaría del Medio Ambiente y Recursos Naturales. All the males showed distinctive reproductive conditions, such as intense ventral colouration, development of femoral pores (Martínez-Torres *et al.*, 2019b) and *in situ* courtship behaviors. We obtained morphometric values such as snout–vent length (SVL) (using digital Vernier calipers to the nearest 0.01 mm) and body weight for each individual (using a digital scale at 0.1 g precision). We kept them in outdoor enclosures (3.0 × 5.0 × 2.0 m) in the FESI-UNAM greenhouse, with free access to water and food (mealworms, grasshoppers and crickets). Furthermore, we released lizards into their incoming habitat after the experimental procedures.

### Semen collection

We induced relaxation in each male with sodium pentobarbital intracelomically (16 mg/kg) (Martínez-Torres *et al.*, 2019a). Immediately after reaching the unconscious state, we cleansed the cloaca with the reptile’s physiological saline solution (0.7%) and obtained semen by pressing the genital papillae. We measured volume and sperm concentration according to Martínez-Torres *et al.* (2019b). All procedures were conducted with the approval of the Institutional Subcommittee for the Care and Use of Experimental Animals of the Faculty of Veterinary Medicine and Zootechnics (UNAM): MC-2018/2-16.

### Cryopreservation and thawing

The ejaculates were diluted up to a final volume of 150 μl with TEY medium (211 mM TES, 96 mM Tris, 11 mM dextrose, 1% penicillin–streptomycin in 20% fresh chicken egg yolk, pH 7.6, 323 mOsm). We employed one part of the dilution (50 μl) for quality assessment and cooled the remaining 100 μl to 5°C at a rate of 0.1°C/minute. We added 100 μl 16% glycerol TEY medium (v/v) in two 10-minute incubation steps to reach a final concentration of 8% glycerol (Young *et al.*, 2017). Subsequently, we made 40 μl pellets on solid CO_2_ (−78°C) and kept them for 2 minutes before immersion in liquid nitrogen for at least 3 weeks of storage. To thaw sperm, we incubated it for 3 minutes at 29°C and diluted the samples (1,1, v/v) with TEY. The fact that incubation at >30°C produced immotile sperm (unpublished data) allowed us to choose this temperature. The sperm assessments were performed in triplicate.

### Sperm assessment

Progressive motility, the percentage of sperm showing forward movement, was observed under light microscopy (Leica DM100) with a 40× objective (Martínez-Torres *et al.*, 2019b). We evaluated the percentage of viability and normal morphology after staining with eosin-nigrosin; we used SYBR-14/propidium iodide (PI) staining (LIVE/DEAD Sperm Viability Kit: Thermo Fisher Scientific) to assess the integrity of the plasma membrane and PSA-FITC lectin staining (0770, Sigma, St. Louis MO, USA) using PI as a contrast for the acrosome integrity according to Alcántar-Rodriguez and Medrano (2017). We expressed all results as a percentage.

### Cryoresistance ratios and sperm quality index

The cryoresistance ratios in each individual were obtained as reported by O’Brien *et al.* (2019) [(after thawing value/before thawing value) × 100] for every sperm assessment. The sperm quality index was obtained as the mean of all the ratios to evaluate the response to cryopreservation.

**Table 1:** Morphometric and semen characteristics of males of the genus *Sceloporus*

Species	Body weight (g)	SVL (cm)	Number of emissions	Semen volume (μl)	Sperm concentration (× 10 ^6^/ml)
*S. aeneus* (*n* = 21)	3.46 ± 0.58^a^ (2.61–4.76)	4.91 ± 0.47^a^ (4.20–5.60)	2.61 ± 0.74 (1.0–4.0)	3.90 ± 1.88^a^ (1.5–9.0)	223.39 ± 133.74 (62.40–535.00)
*S. grammicus* (*n* = 20)	11.41 ± 2.74^b^ (5.52–15.27)	7.07 ± 0.6^b^ (6.0–8.50)	2.95 ± 1.14 (1.0–5.0)	5.92 ± 3.72^a^ (1.0–16.0)	285.24 ± 190.19 (79.68–780.0)
*S. torquatus* (*n* = 21)	33.20 ± 9.29^c^ (18.07–52.48)	9.79 ± 0.95^c^ (8.0–11.30)	3.04 ± 1.11 (1.0–5.0)	13.57 ± 9.58^b^ (3.0–32.0)	272.84 ± 211.8 (69.56–765.0)

The data presented are the mean ± standard deviation. The data range is presented in parentheses. The significant differences among the species (*P* < 0.05) are represented by different letters (^a,b,c^).

### Statistical analysis

We transformed the data with arcsin, and the values are presented as the mean ± standard deviation. Assumptions of normality and homogeneity of variance were tested using the Shapiro–Wilk test or Levene’s test, respectively. We performed Pearson analysis to determine the correlations between semen volume, sperm concentration and morphometric values in each species. Significant differences among species were determined using one-way ANOVA or the Kruskal–Wallis test followed by a multiple comparison (Holms–Sidak or Dunn, respectively) for morphometric, fresh and post-thawing sperm values, cryoresistance ratios and sperm quality indices. The effect of sperm cryopreservation was analyzed by either the *t*-test or Wilcoxon test. A *P*-value of less than 0.05 was considered statistically significant. We carried out all tests using the SigmaPlot 10 program for Windows.

## Results

### Animal and semen characteristics

We obtained semen from all males (*n* = 62) used. There were significant differences among species in SVL (H _(2)_ = 53.91, *P* < 0.001) and body weight (H _(2)_ = 54.22, *P* < 0.001). The mean values, standard deviations and intervals of these characteristics are presented in Table 1. The appearance of fresh semen in all organisms was milky white, with a thick consistency, absent of feces, urates and blood cells. No significant differences were found either in the number of emissions (F_(2, 61)_ = 1.024, *P* = 0.365) or the sperm concentration among species (H _(2)_ = 0.791, *P* = 0.673). *Sceloporus torquatus* provided a significantly greater volume (13.5 ± 9.5 μl) than the other species (H _(2)_ = 19.67, *P* < 0.001).

### Correlation between morphometrics of male individuals and semen

We found positive correlations between body weight and semen volume in *S. grammicus* (*r* = 0.634, *P* = 0.002) and *S. torquatus* (*r* = 0.507, *P* = 0.019) and a negative correlation between body weight and sperm concentration in *S. grammicus* (*r* = −0.574, *P* = 0.008). There was no correlation between semen volume and sperm concentration in any species (*P* > 0.05; Table 2).

**Table 2:** Correlation between seminal values and morphometrics values of males of the genus *Sceloporus*

Species	Body weight and semen volume	SVL and semen volume	Body weight and sperm concentration	SVL and sperm concentration	Semen volume and sperm concentration
*S. aeneus*	*r* = 0.091 *P* = 0.694	*r* = 0.071 *P* = 0.759	*r* = 0.111 *P* = 0.630	*r* = −0.199 *P* = 0.386	*r* = −0.407 *P* = 0.066
*S. grammicus*	*r* = 0.634 *P* = 0.002^*^	*r* = 0.427 *P* = 0.060	*r* = −0.574 *P* = 0.008*	*r* = −0.417 *P* = 0.671	*r* = −0.340 *P* = 0.132
*S. torquatus*	*r* = 0.507 *P* = 0.019^*^	*r* = 0.199 *P* = 0.387	*r* = −0.286 *P* = 0.209	*r* = −0.205 *P* = 0.373	*r* = −0.407 *P* = 0.066

^*^Significant correlations (*P* < 0.05).

### Sperm assessment before and after cryopreservation

**Figure 1:**
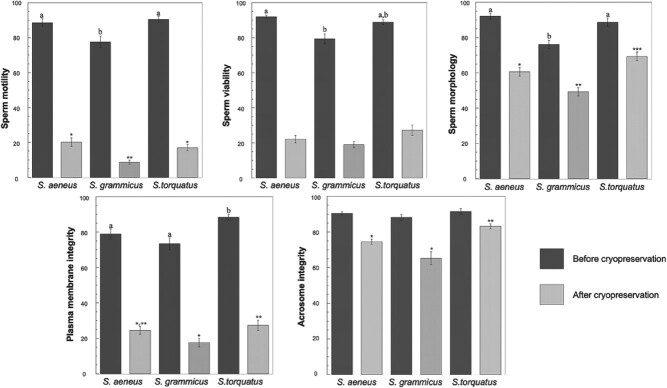
Changes on sperm of Sceloporus lizards before and after cryopreservation. The lines represent the standard deviation. The significant differences between the means (*P* < 0.05) are indicated by different literals.

We found significant differences among species in every parameter before cryopreservation (*P* < 0.05), except in acrosome integrity (*P* = 0.243). The freeze–thaw process produced changes that significantly decreased the sperm quality of the species studied (*P* < 0.05). After thawing, there were significant differences among species, except for sperm viability (H _(2)_ = 17.19, *P* = 0.099) (Fig. 1).

### Cryoresistance ratios

Cryoresistance ratios showed significant differences among species only for sperm motility (H _(2)_ = 13.71, *P* = 0.001), morphology (H _(2)_ = 7.88, *P* = < 0.001) and acrosome integrity (H _(2)_ = 15.26, *P* = < 0.001). The sperm quality index showed a better response in *S. torquatus* and *S. aeneus* compared with *S. grammicus* (F _(2, 59)_ = 8.84, *P* < 0.001) (Table 3).

**Table 3:** Sperm cryoresistance for the different variables studied in individuals of the genus *Sceloporus*

Species	Sperm motility	Sperm viability	Sperm morphology	Membrane integrity	Acrosome integrity	Sperm Quality Index
*S. aeneus*	22.93 ± 12.56^a^	24.00 ± 10.46	65.88 ± 12.53^a^	31.63 ± 13.31	82.64 ± 7.90^a^	45.42 ± 7.75^a,b^
*S. grammicus*	11.12 ± 6.45^b^	24.37 ± 10.69	65.21 ± 12.88^a^	24.08 ± 14.00	74.01 ± 17.08^a^	39.75 ± 7.99^a^
*S. torquatus*	18.80 ± 8.48^a^	30.79 ± 15.35	78.45 ± 10.88^b^	31.82 ± 16.68	91.31 ± 8.21^b^	50.23 ± 8.19^b^

^a,b,c^ = The significant differences between the means (*P* < 0.05) are indicated with different letters.

## Discussion

Gamete cryopreservation is a tool with great potential to complement conservation programs and can be successfully used to reduce the loss of lizard diversity (Clulow and Clulow, 2016; Perry and Mitchell, 2021). Although it allows the storage of genomes from threatened species, there are scarcely cases where all of them have used destructive techniques to obtain semen (Young *et al.*, 2017; Campbell *et al.*, 2020, 2021). In this study, we developed a non-invasive method for sperm cryopreservation in sceloporine lizards and compared the responses among the studied species.

### Semen characteristics

We obtained samples of excellent appearance, as described by other authors (Zimmerman *et al.*, 2013; López-Juri *et al.*, 2018; Martínez-Torres *et al.*, 2019b). However, the volumes of ejaculates were higher than those reported previously for the same species, possibly because we isolated males before reproductive season (Martínez-Torres *et al.*, 2019a). *Sceloporus torquatus* volumes were also higher than those of the other species. The above may be due to the difference in testicular volume among species (Ramírez-Bautista *et al.*, 2012; Sánchez Rivera, 2017), since lizards lack accessory glands to compose the semen (Rheubert *et al.*, 2014) and the correlation we found between semen volume and body weight. Regarding sperm concentration, there were no significant differences among species. There was no relationship between semen volume and sperm concentration, but we found a negative correlation between sperm concentration and body weight in *S. grammicus*. Multiple factors can modify the sperm count in lizards, such as diet (Kahrl and Cox, 2015), temperature, photoperiod or contaminants in the environment (Hall and Warner, 2017, 2018). Moreover, in several wild species, including lizards, it has been reported that sperm concentration is unrelated to body mass (Comizzoli *et al.*, 2012; Hobbs *et al.*, 2021).

### Sperm cryopreservation

We found that sperm motility, viability, normal morphology, plasma membrane and acrosome integrity diminished with the freeze–thaw process (Yeste, 2016). Similar findings have been reported in other reptile species, such as *Crocodylus porosus* (Johnston *et al.*, 2014b), *Crotalus ruber* (Zacariotti *et al.*, 2012), *Tupinambis merianae* (Young *et al.*, 2017) and *Varanus panoptes* (Campbell *et al.*, 2020). We tested the 8% glycerol TEY medium since it had the best response for this cryoprotectant agent (CPA) in another lizard (Young *et al.*, 2017). Despite advances with other CPAs, it should be evaluated for sceloporine lizard sperm.

Maintaining a high rate of sperm motility after cryopreservation is essential for sperm migration and overcoming physiological barriers to fertilization (Dcunha *et al.*, 2022). Therefore, for the recovery of sperm motility, the selection of samples must be adequate. Sperm motility is high in lizards (>75%) (Zimmerman *et al.*, 2013; López-Juri *et al.*, 2018; Martínez-Torres *et al.*, 2019b), but the time of collection is also relevant since this characteristic decreases as the reproductive season progresses (Depeiges and Dacheux, 1985; Martínez-Torres *et al.*, unpublished data). Low-percentage samples (<40%) are commonly discarded for ART procedures (as occurred with some individuals of S*. grammicus* outside the mating season) but should be considered for rational management if it is the only resource for the endangered species.

Several factors influence the recovery of sperm since exposure to potential damage, such as osmotic and thermal stress, occurs during the freezing stages. We suggest that the optimal extender composition and cooling rates should be explored to diminish these effects in each species (Varisli *et al.*, 2013). Since the use of TEY in reptiles has been shown to be beneficial (Young *et al.*, 2017), we suggest focusing efforts on determining the optimal cooling rate, as in our case, it may be too slow to accentuate the effect of dehydration during the freezing process (Santiago-Moreno and Galarza, 2019). Young *et al.* (2017) found the best response when cooling lizard sperm at a 0.3–1°C/min rate regardless of the type and amount of CPA. The use of additives in cryopreservation protocols should also be considered, as they can increase mobility (Ezzati *et al.*, 2019; Bahmyari *et al.*, 2020; Campbell *et al.*, 2021).

Sperm morphology did not show abnormalities before freezing. We only found a cytoplasmic droplet near the neck region of sperm (Johnston *et al.*, 2014a); this structure is considered to be a source of endogenous energy rather than an indicator of immaturity in reptiles (Zhang *et al.*, 2015). However, future work is needed to confirm this supposition. Most of the abnormalities (swelling and rupture of spermatozoa) occurred after cryopreservation. This could be associated with the freezing process since the formation of ice crystals produces irreversible morphological changes (Ozkavukcu *et al.*, 2008). Although Escalier and Bisson (1980) reported that one of the most susceptible organelles during freezing–thawing is the acrosome, this structure was the least affected in our study, as seen Young *et al.* (2017).

Particularly, the plasma membrane integrity is relevant to assess considering its relevance to metabolic exchange, sperm capacitation, acrosomal reaction, union and fusion with oolemma (Brito *et al.*, 2003). In mammals, the specific composition of lipids is related to sperm quality. Macías García *et al.* (2011) observed a significant correlation between a high saturation of phospholipids and poor sperm quality. In contrast, highly polyunsaturated fatty acids are related to the proportion of intact sperm (Tapia *et al.*, 2012). We found a higher tolerance to freezing in *S. torquatus* and *S. aeneus* than in *S. grammicus*. It is possible that these factors contribute to the lower resistance of *S. grammicus*. The increase in the percentage of cryoprotectants improves this characteristic in some reptiles but may impair sperm motility (Johnston *et al.*, 2014b; Young *et al.*, 2017). This complex interaction needs to be explored for sceloporine lizards. Knowledge about the membrane composition may also benefit the design of media components but is still scarce for lizard sperm.

Since sperm cryotolerance varies among species and individuals, the media and CPA choice are of the utmost importance to preserve the functional and survival characteristics of gametes. It is necessary to establish species-specific conditions according to the response to the procedure, as is done routinely in mammals (Contreras-Méndez and Medrano, 2016; Alcántar-Rodriguez and Medrano, 2017). Individual identification based on sperm cryotolerance (establishing good and poor freezers) may also allow the management of different genetic resources to increase survival and genetic diversity for ART implementation (Yeste, 2016; O’Brien *et al.*, 2019).

## Conclusion

The TEY medium supplemented with 8% glycerol allowed sperm cryopreservation in sceloporine lizards. However, this methodology produced a significant diminution of sperm quality. Further work is planned to optimize the composition of the diluents, the choice and amount of cryoprotectant and the cooling–thawing rates and to consider the employment of additives in each species to improve sperm cryosurvival.

## Funding

This work was supported by the Universidad Nacional Autónoma de México PAPIIT project (IN220419, IN205421) and the Consejo Nacional de Ciencia y Tecnología scholarship (703732) awarded to U.Á.S.R.

## Supplementary Material

suppl_coac068
