# Supplementary material for: Implementation of a method for sperm cryopreservation in sceloporine lizards
Source: Conserv Physiol. 2022 Nov 9;10(1):coac068. doi: 10.1093/conphys/coac068 (PMC9644977; doi:10.1093/conphys/coac068)
Supplement: suppl_coac068 [file suppl_coac068.zip › Supplementary Figure.pdf]

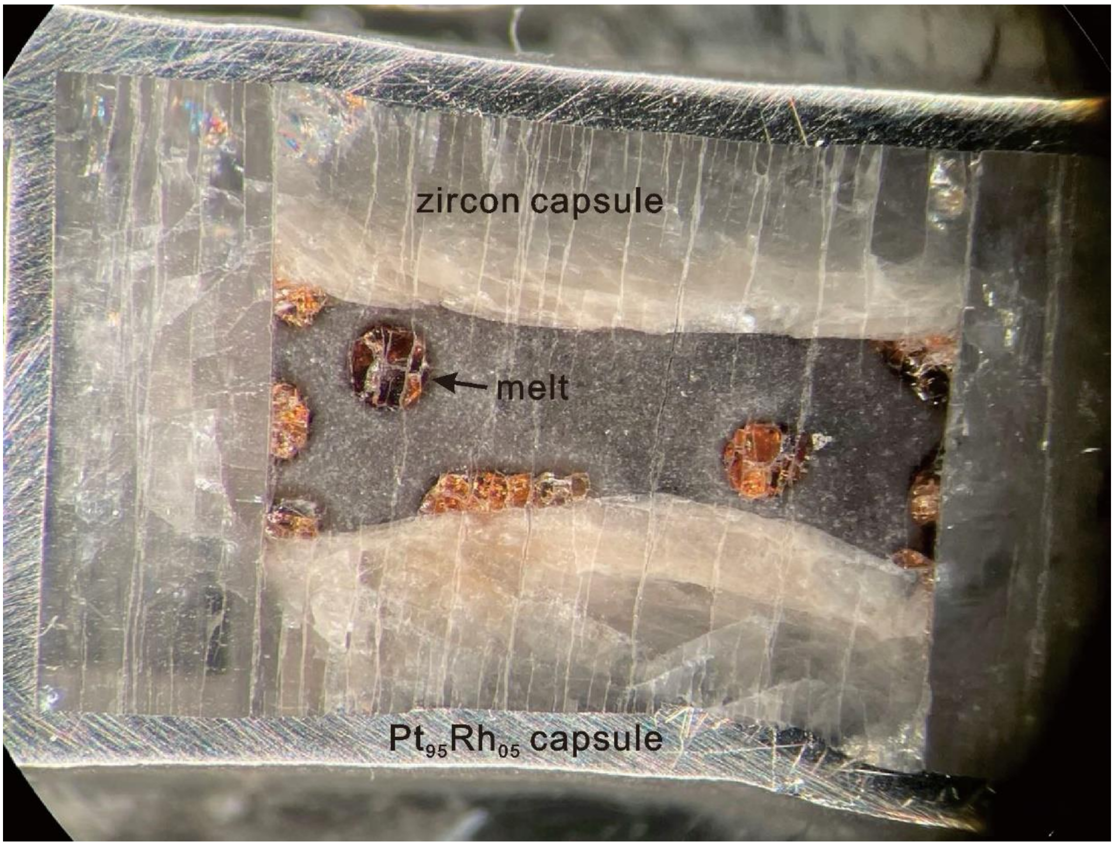

Figure S1 Half-polished capsule photo of run JZ10, showing the noble metal capsule, zircon liner, and the produced melt around the garnet megacryst. As can be seen, the zircon liner provides a tight seal that efficiently prevents reaction of the starting material with the $Pt_{95}Rh_{05}$ capsule.
